# Umbilical Artery Thrombosis with Associated Acute and Severe Fetal Growth Restriction and Transient Severe Protein S Deficiency: Report of a Case with Prenatal Ultrasound Diagnosis Allowing for Timely Intervention and Good Outcome

**DOI:** 10.1155/2018/6324362

**Published:** 2018-07-09

**Authors:** Ali Alhousseini, Sunil Jaiman, Edgar Hernandez-Andrade, Salam Zeineddine, Faisal Qureshi, Suzanne M. Jacques

**Affiliations:** ^1^Department of Obstetrics and Gynecology, Wayne State University, Detroit, MI, USA; ^2^Department of Physiology, Wayne State University, Detroit, MI, USA; ^3^Department of Pathology, Wayne State University, Detroit, MI, USA; ^4^Department of Internal Medicine, Wayne State University, Detroit, MI, USA

## Abstract

**Background:**

Thrombosis of one of the umbilical arteries can be associated with adverse pregnancy outcomes such as stillbirth and severe intrauterine growth restriction (IUGR).

**Case:**

A 21-year-old gravida 1 patient, with a history of 3-vessel cord at 20 weeks, presented at 29 weeks with a single umbilical artery. The estimated fetal weight measurements at 26 weeks, 29 weeks, and 31 weeks were at the 27th percentile, the 26th percentile, and less than the 5th percentile, respectively. At 33 weeks, amniotic fluid index became abnormal at 2.3 cm and fetal heart tracing revealed spontaneous prolonged decelerations, and a cesarean delivery was performed. Placental pathology showed thrombosis of one of the umbilical arteries. At birth, a transient protein S deficiency was detected (activity 13%) and resolved at two months of age (activity 66%). The baby had an uneventful clinical course since birth.

**Conclusion:**

The recognition of reduction of umbilical arteries from two to one allowed for timely intervention with good outcome in this case. Thrombosis of umbilical vessels may be associated with a deficiency in coagulation proteins such as protein S.

## 1. Introduction

The prenatal diagnosis of the reduction of umbilical arteries from two to one has been associated with adverse perinatal outcomes such as stillbirth and intrauterine growth restriction (IUGR) [1–5]. The ability to utilize ultrasound imaging to diagnose the reduction of number of functional umbilical arteries may help in avoiding serious adverse perinatal outcomes such as stillbirth.

## 2. Case

**Figure 1:**
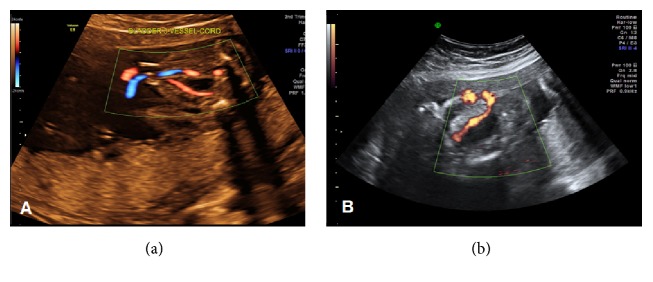
Cross-sectional images of the fetal pelvis: (a) at 20 weeks of gestation showing with color Doppler the two umbilical arteries around the fetal bladder and (b) at 29 weeks of gestation with only one umbilical artery.

**Figure 2:**
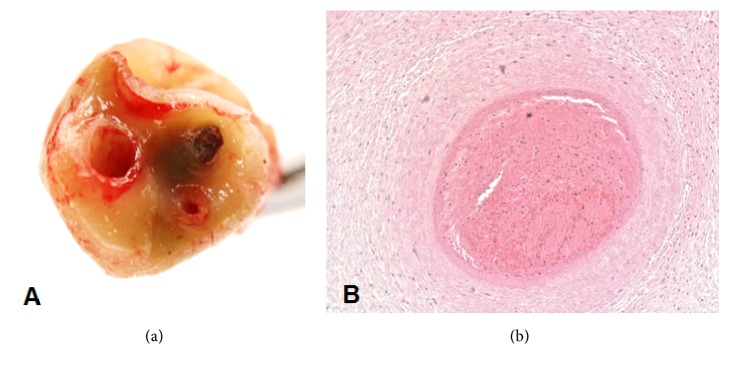
Cross-sectional images of the umbilical cord (gross (a) and histologic (H&E) (b)) showing thrombosis of one of the umbilical arteries.

A 21-year-old gravida 1 para 0 patient presented at 20 weeks for a routine anatomy scan that revealed normal anatomy survey including two umbilical arteries (Figure 1(a)). She had an unremarkable past medical history. The estimated fetal weight was 874 grams (27th percentile) at 26 weeks and 1306 grams (26th percentile) at 29 weeks. Ultrasound at 29 weeks revealed a single umbilical artery (Figure 1(b)) raising the suspicion for a pathological process. Fetal echocardiogram was normal. At 31 weeks, the estimated fetal weight was 1349 grams (less than 5th percentile) with normal Doppler evaluation and biophysical profile. At 32 weeks, the amniotic fluid index was 10.1 cm, the biophysical profile was 10/10, and the cerebroplacental ratio decreased to 1.083. At 33 weeks, the amniotic fluid index decreased to 2.3 cm, maternal and fetal Doppler evaluations were normal except for abnormal cerebroplacental ratio of 1.08, and fetal heart tracing showed spontaneous prolonged decelerations. Secondary to the recurrent prolonged decelerations, a primary cesarean delivery was performed, and a live male infant was delivered weighing 1395 g with APGAR scores of 8 and 9 at 1 and 5 minutes, respectively. Placental pathology showed thrombosis of one of the umbilical arteries with necrosis of the medial myocytes (Figures 2(a) and 2(b)). It also showed subendothelial fibrin deposition in stem villous blood vessels, chorionic villous hypervascularity, and a small subchorionic placental infarct. At birth, the baby had no signs related to thrombosis. Thrombophilia profiles showed a severe protein S deficiency (activity 13%) at birth which resolved at two months of age (activity 66%). The neonate has an uneventful clinical course since birth.

## 3. Discussion

This case demonstrates the importance of the prenatal ultrasound diagnosis of the reduction in umbilical arteries to one artery, allowing for a close follow-up and delivery at 33 weeks of gestation, thus avoiding adverse perinatal outcomes such as stillbirth. Heifetz [1] estimated that thrombosis of the umbilical cord occurs in approximately 1 in 1300 deliveries and 1 in 1000 perinatal autopsies, and that umbilical artery thrombosis carries a worse prognosis than venous thrombosis. Sato and Benirschke [5] evaluated 11 cases with umbilical artery thrombosis, reporting two with stillbirths and three with severe intrauterine growth restriction. The authors proposed that occlusive thrombi of the umbilical arteries led to the necrosis of the inner layer of the media because of the lack of vasa vasorum. This is consistent with our case. Shilling et al. [2] performed a retrospective study evaluating thrombosis of one of the umbilical arteries in five placentas and in two autopsies over a 13-year period (an estimated 116,000 deliveries). All seven cases had poor outcomes including two stillbirths, three with intrauterine growth restriction, one with caudate infarction, and one with schizencephaly and partial acrania.

Newborn infants have a reduction of synthesis of several coagulation proteins including protein S, protein C, and antithrombin [6]. The likelihood of thrombotic complications during the first month of life is 40 times higher than at any other pediatric age [6]. This reduction is more pronounced in preterm infants and would require around 3 months to reach normal values [6]. These findings are consistent with our case with very low protein S activity at birth and normalization at two months of life.

This case demonstrates that prenatal diagnosis of umbilical artery thrombosis was a crucial step in avoiding possible serious adverse perinatal outcome [1–4]. Thrombophilia work-up of future cases may shed light on the association between this serious thrombotic event and an underlying thrombophilia such as protein S deficiency. We speculate that this deficiency was present before delivery and may have played a role in the thrombosis of one of the umbilical arteries.
